# Prepancreatic postduodenal portal vein: a case report and literature review

**DOI:** 10.1186/s40792-023-01644-5

**Published:** 2023-04-23

**Authors:** Masanori Akashi, Daiki Miyazaki, Kazuaki Hashimoto, Shogo Fukutomi, Shoichiro Arai, Yuichi Goto, Toshihiro Sato, Hisamune Sakai, Toru Hisaka

**Affiliations:** Department of Surgery, Kurume University School of Medicine, 67 Asahi-Machi, Kurume, Fukuoka 830-0011 Japan

**Keywords:** Anomaly of the portal venous system, Prepancreatic postduodenal portal vein, Hepatectomy, Hepatocellular carcinoma

## Abstract

**Background:**

Among congenital anomalies of the portal venous system, prepancreatic postduodenal portal vein (PPPV) is very rare and has only been reported to date. Herein, we report a case of PPPV identified in preoperative examinations for hepatocellular carcinoma and a literature review.

**Case presentation:**

A 63-year-old man was admitted to our hospital for treatment of a liver tumor. After examination, he was diagnosed with hepatocellular carcinoma with a diameter of 40 mm in segment 8. Contrast-enhanced computed tomography scan showed a portal vein passing between the duodenum and pancreas, hence called PPPV. At the hepatic hilus, the portal vein branched off in a complicated course with some porto-portal communications. We determined that anatomical resection with manipulation of the hepatic hilum in this case resulted in major vascular injury. Therefore, we performed partial liver resection, and the patient was discharged uneventfully on postoperative day 14.

**Conclusions:**

Although PPPV is an extremely rare congenital vascular variant, it is important to carefully identify vascular patterns preoperatively and to recognize the possibility of such an anomaly to avoid misidentification and inadvertent injuries during surgery.

## Background

Although the frequency of portal system anomalies is lower than that of the bile ducts and arteries, its anatomical recognition is very important for safe gastrointestinal surgery, especially hepatobiliary–pancreatic surgery. Among congenital malformations of the portal system, the preduodenal portal vein (PDPV) is occasionally reported as a comorbidity of other visceral malformations [1, 2]; however, the prepancreatic postduodenal portal vein (PPPV) is extremely rare. Here, we report a case of hepatocellular carcinoma with a PPPV malformation and a complex branching of the portal vein at the hepatic hilus, and review the literature reported to date.

## Case presentation

A 63-year-old man complaining of discomfort in the upper right abdomen was referred to our hospital because of a liver tumor diagnosed by another hospital.

Previously, he had undergone an appendectomy, and had a history of untreated chronic hepatitis C and a daily habit of drinking. His physical examination revealed no notable abnormalities. The abnormal values in his blood test findings were as follows: aspartate transaminase, 71 IU/l; alanine transaminase, 108 IU/l; alpha-fetoprotein, 5626.0 ng/ml; and protein induced by vitamin K absence or antagonist II, 642 mAU/ml. Other blood counts, biochemical laboratory findings and coagulation factors were within the normal ranges. The retention rate of indocyanine green at 15 min was 10%.

**Fig. 1:**
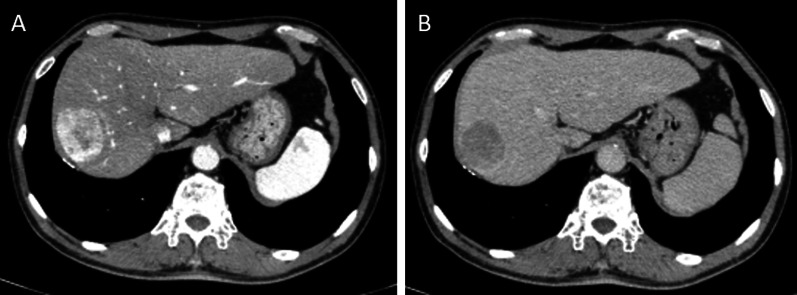
Contrast-enhanced computed tomography findings (focused on tumor). **A** Early phase. **B** Delayed phase. A 40-mm tumor in segment 8 of the liver, which was enhanced in the early phase and washed out in the delayed phase, suggesting hepatocellular carcinoma

**Fig. 2:**
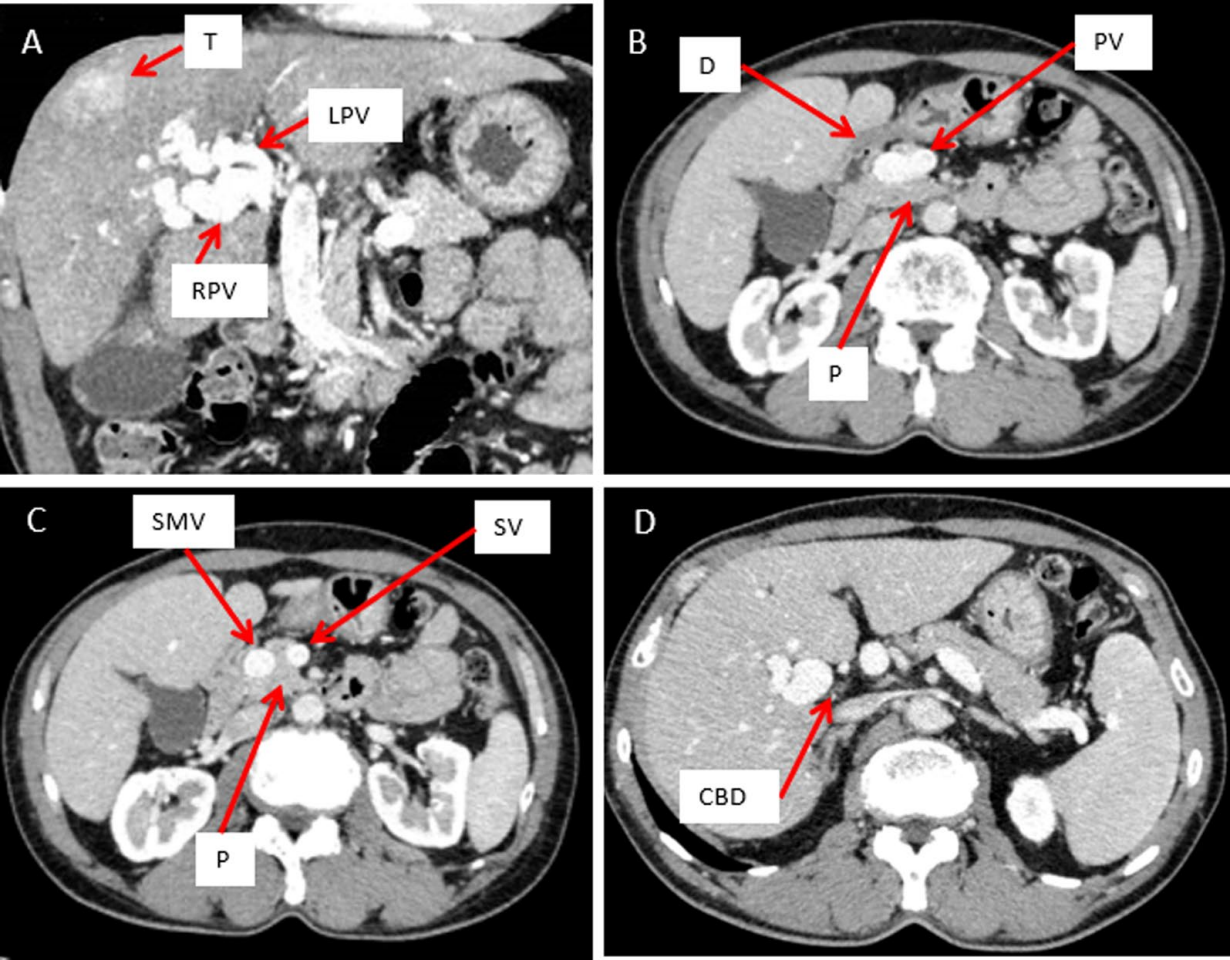
Contrast-enhanced computed tomography findings (focused on portal vein). **A** Coronal view. Portal vein dilated in the hepatic hilus and bent and twisted while branching off. **B**, **C** Portal vein was lying ventral to the pancreas and dorsal to the duodenum. **D** Portal vein was lying ventral to the common bile duct. *T* tumor, *RPV* right portal vein, *LPV* left portal vein, *D* duodenum, *PV* portal vein, *P* pancreas, *SMV* superior mesenteric vein, *CBD* common bile duct

**Fig. 3:**
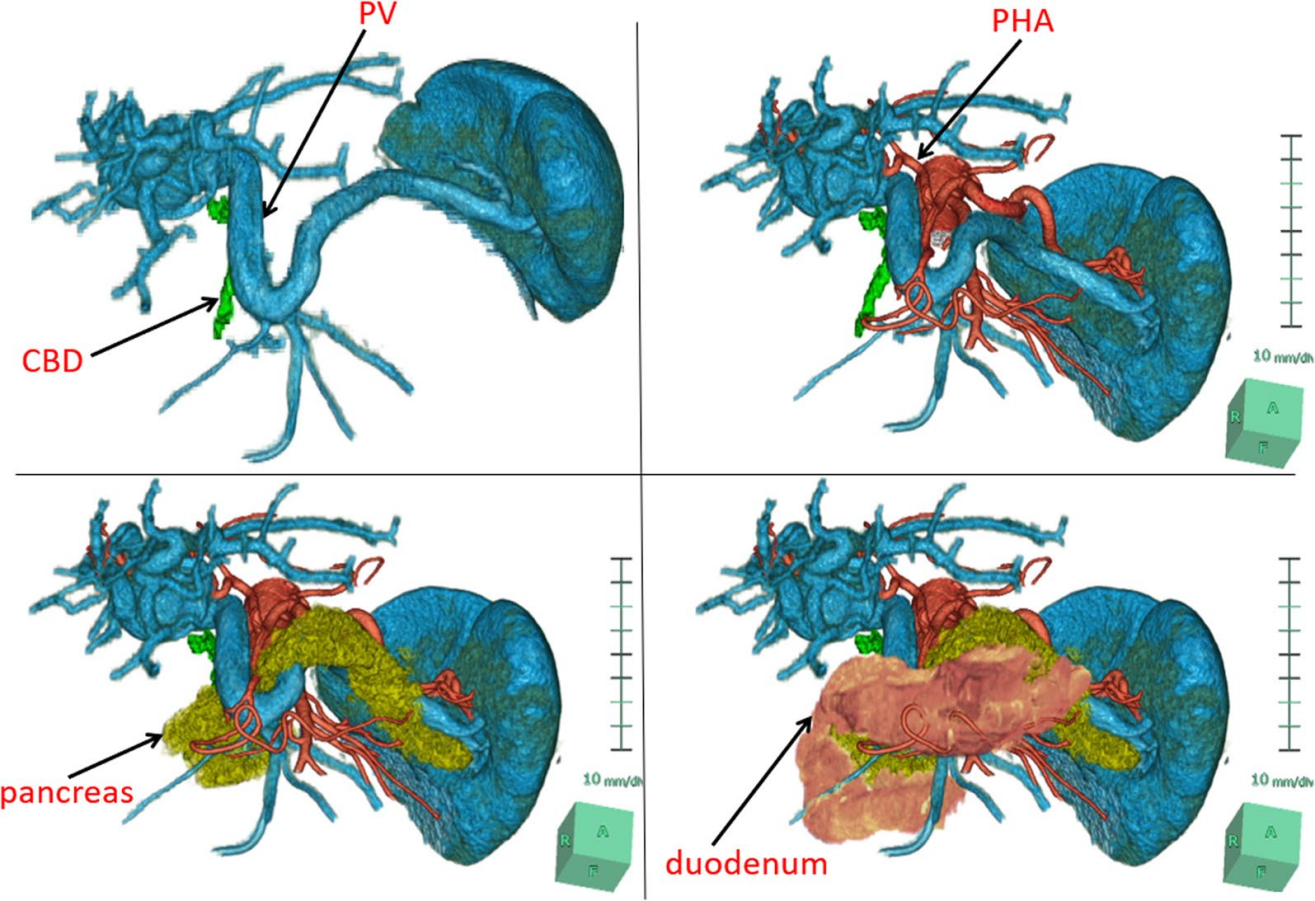
Volume rendering 3D reconstruction of computed tomography. The image shows the portal vein running a prepancreatic postduodenal course, lying in front of the common bile duct, and forming an inverted L-shape, convexly caudad. *CBD* common bile duct, *PV* portal vein, *PHA* proper hepatic artery

**Fig. 4:**
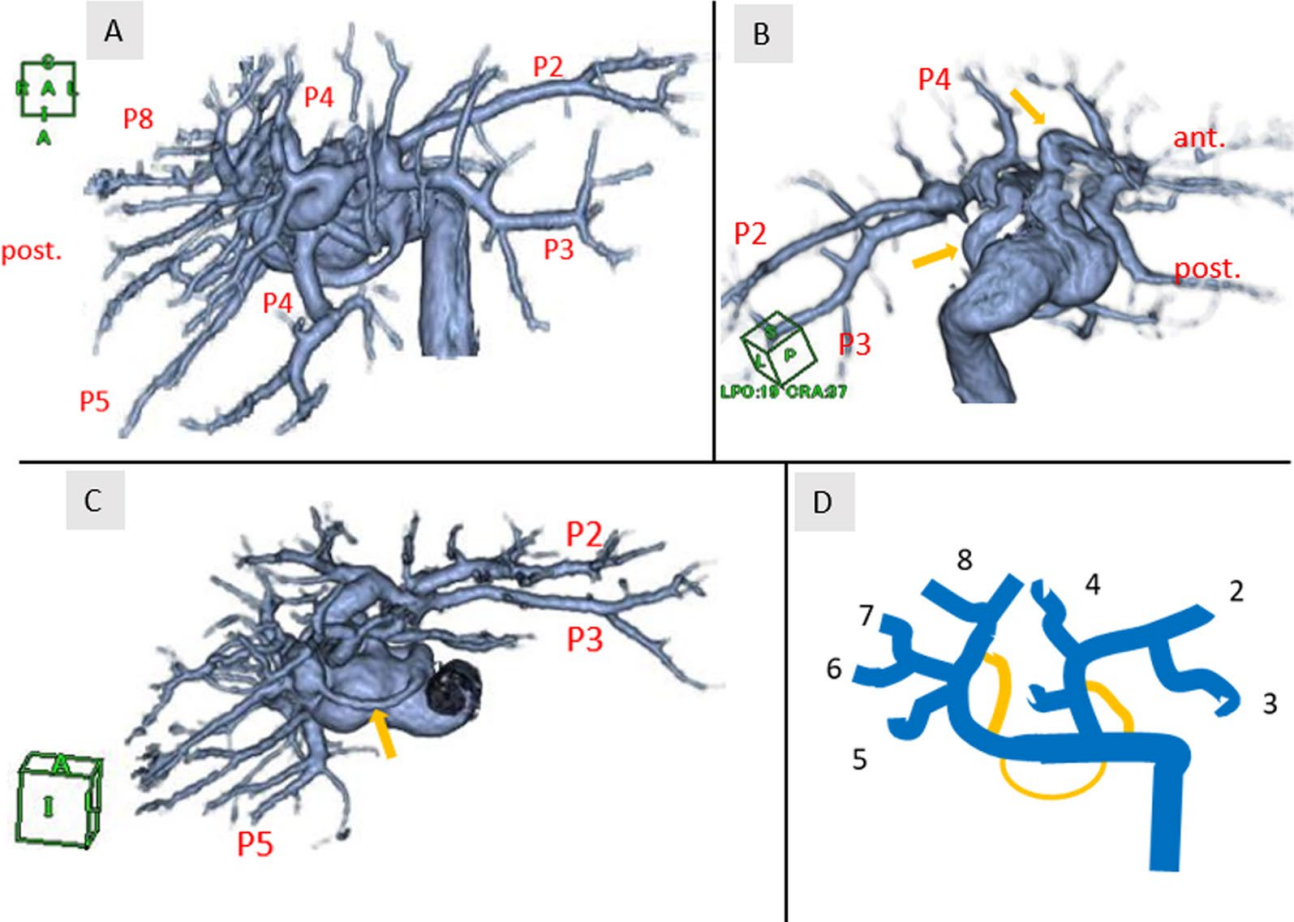
**A**–**C** Volume rendering 3D reconstruction of computed tomography (focused on hepatic hilus). The image shows the complex branching pattern of the portal vein and some porto-portal communications (yellow arrow). **D** Scheme illustrates portal vein branching and the yellow line indicates porto-portal communications

Contrast-enhanced computed tomography (CT) showed a 40-mm tumor in segment 8 (S8) of the liver, which was enhanced in the early phase and washed out in the delayed phase, suggesting hepatocellular carcinoma (HCC) (Fig. 1). His portal vein was lying ventral to the pancreas and dorsal to the duodenum (hence called PPPV), and ventral to the common bile duct (Figs. 2, 3). In the hepatic hilus, the portal vein was dilated, forming an inverted L-shape, and was branching while winding with an irregular caliber (Figs. 2A, 4). In addition, there were some porto-portal communications (Fig. 4). No anomalies were detected in the common bile duct, gallbladder, hepatic artery, and there were no esophageal or gastric varices, thrombus, and portosystemic collaterals.

**Fig. 5:**
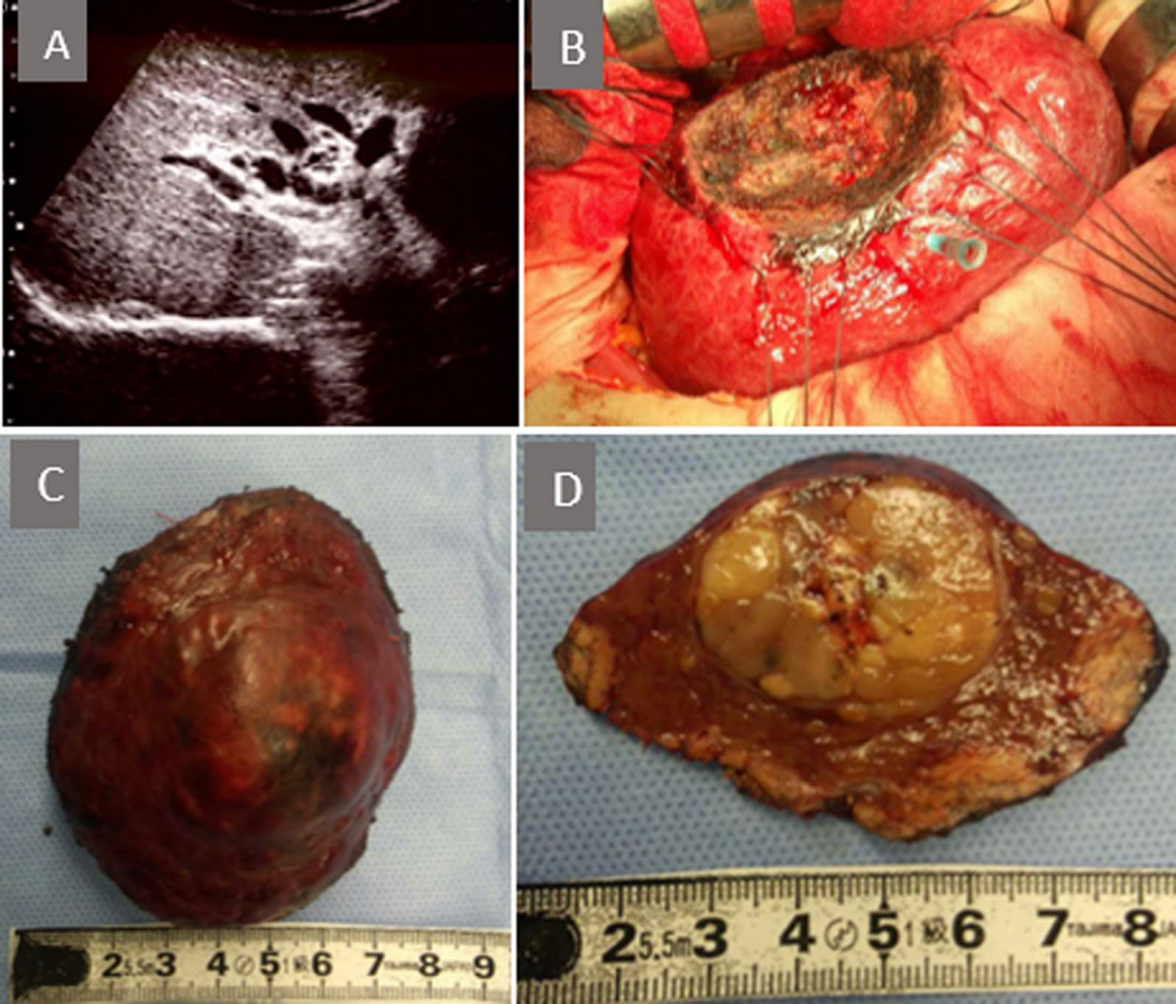
**A** Intraoperative ultrasonography showed the complex branching of the portal vein in hilus of the liver. **B** Liver partial resection was performed. **C**, **D** Resected specimen

Because of this anomalous configuration, the Glissonean approach at the hepatic hilum or anatomical resection was judged to be dangerous; therefore, we performed partial resection of S8. During the operation, the liver had a chronic hepatitis pattern and ascites was not observed. No morphological malformations were detected in the liver or in other organs. Intraoperative ultrasonography showed worm-like meandering of the intrahepatic portal veins, but no occlusion findings due to a thrombus (Fig. 5A). We performed liver resection with a margin of 2 cm from the tumor without the Pringle maneuver (Fig. 5B–D). The operative time was 431 min, and the amount of blood loss was 785 ml. The final pathological diagnosis was moderately differentiated hepatocellular carcinoma with an UICC classification of pT2N0M0, Stage II. The fibrosis stage and inflammatory grade of the resected liver were both F3A2 according to the New Inuyama classification. The postoperative course was uneventful, and the patient was discharged 14 days after the surgery.

## Discussion

**Fig. 6:**
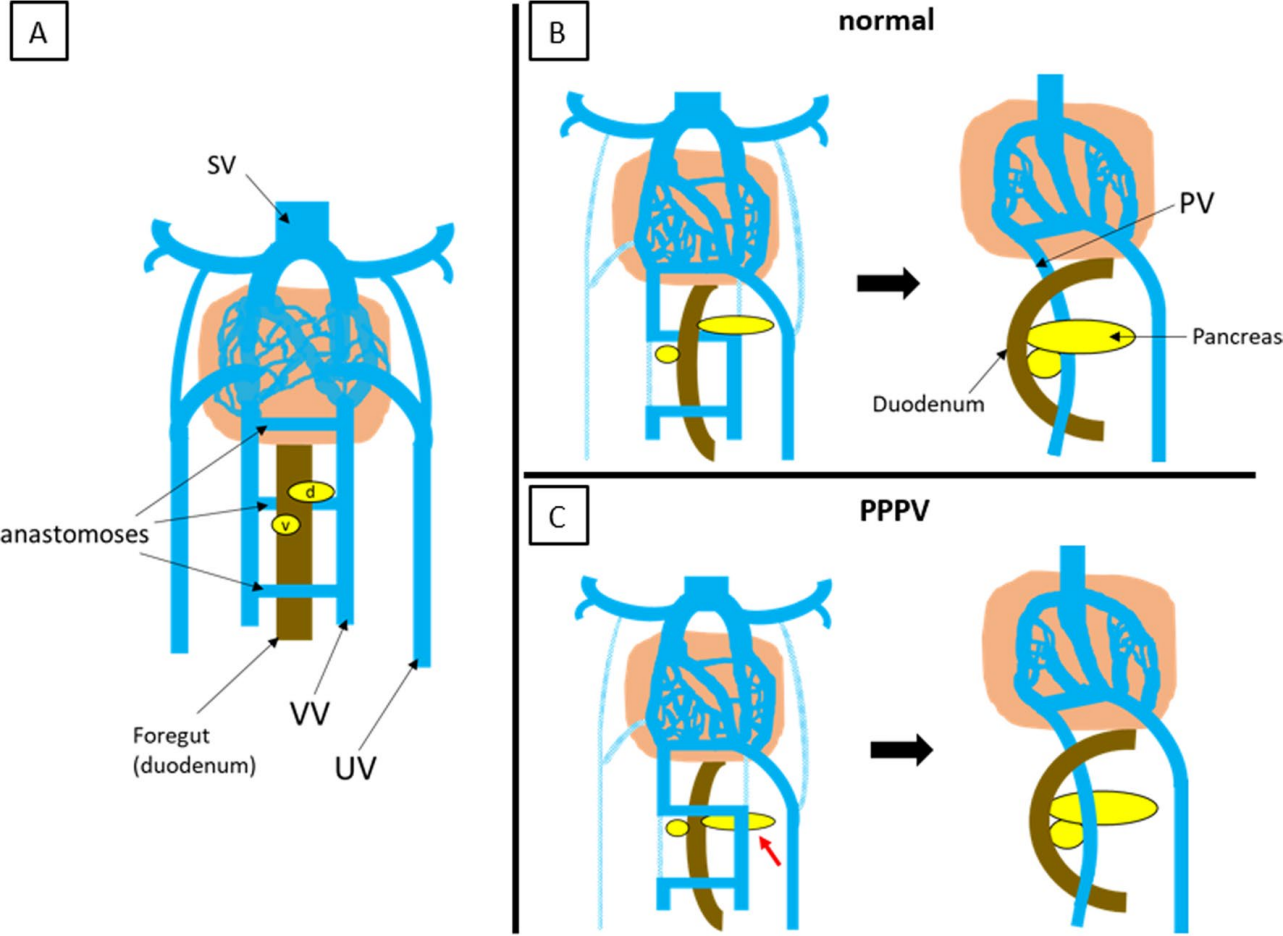
Development of the portal vein. **A** Paired vitelline veins are connected by three anastomoses. The duodenum lies ventral to the intermediate anastomosis. The dorsal pancreatic bud arises ventrally to the left vitelline vein, and the ventral pancreatic bud arises contralateral and slightly caudal to the dorsal pancreatic bud. **B** Normal development of portal vein and pancreas. The lower part of the right vitelline vein and the upper part of the left vitelline vein disappear, and the intermediate anastomosis on the dorsal side of the duodenum forms the portal vein main trunk. As the duodenum rotates, the ventral pancreatic bud fuses behind the dorsal pancreatic bud. **C** Development of the prepancreatic postduodenal portal vein. The dorsal pancreatic bud arises on the dorsal side of the left vitelline vein, or arises caudal to the intermediate anastomosis, which results in the portal vein posterior to the duodenum but anterior to the pancreas. *SV* sinus venosus, *d* dorsal pancreatic bud, *v* ventral pancreatic bud, *VV* vitelline vein, *UV* umbilical vein, *PV* portal vein, *PPPV* prepancreatic postduodenal portal vein

Here, we report a case of PPPV, which is an extremely rare congenital portal vein anomaly. Portal vein development begins at 4 weeks of gestation and is formed by the paired vitelline veins with three anastomoses that traverse the foregut (duodenum). As the weeks pass, the lower part of the right vitelline vein and the upper part of the left vitelline vein disappear, and the intermediate anastomosis on the dorsal side of the duodenum forms the portal vein main trunk [3, 4] (Fig. 6A, B). The development of the pancreas is established by fusion of the dorsal and ventral pancreatic buds arising from the foregut [5, 6]. The dorsal pancreatic bud arises ventrally to the left vitelline vein and the ventral pancreatic bud arises contralateral and slightly caudal to the dorsal pancreatic bud. As the duodenum rotates, the ventral pancreatic bud fuses behind the dorsal pancreatic bud, establishing the normal positional relationship between the portal vein and the pancreatic head (Fig. 6A, B). Regarding the etiology of PPPV, malposition of the dorsal pancreatic bud has been emphasized. Matsumoto et al. hypothesized that PPPV is established when the dorsal pancreatic bud arises on the dorsal side of the left vitelline vein [6]. Tomizawa et al. hypothesized that the formation of the dorsal pancreatic bud caudal to the intermediate anastomosis is the cause of PPPV [7] (Fig. 6C). We summarized 15 PPPV cases reported so far [6–18], including the present case (Table 1). Twelve of these 15 cases have been reported in Japan. In contrast to PDPV, which has been reported as a complication of other visceral malformations in childhood, all cases were adult cases, and only two of the reported cases of PPPV had congenital biliary dilatation, and most of them were reported to have PPPV during examination and treatment for other diseases. In addition to the abnormality in which the portal vein runs in front of the pancreas and behind the duodenum, this disease has the following characteristic findings. The portal vein was L-shaped or inverted L-shaped in ten cases, and in 11 cases, it passed through the ventral or right side of the common bile duct. Five cases with abnormal branching of the portal vein were also reported, most of which involved early branching of the portal vein. Moreover, of the 13 PPPV cases for which information on portal vein morphology was provided, seven (53.8%) had irregularly dilated or winding portal vein in the hepatic hilum, and there were five cases with a fragile and thin portal vein wall and firm adhesion with the surrounding tissue. Five of these patients underwent pancreaticoduodenectomy and three underwent combined resection of the portal vein because of difficulty in dissecting the portal vein [9, 16, 18], and two of them had intraoperatively massive bleeding while isolating the portal vein, and thrombosis in the reconstructed portal vein [16, 18]. Shimizu et al. reported the difficulty of portal vein reconstruction due to the thinness of the portal vein wall and the difficulty of postoperative management due to complications of portal vein thrombosis [16].

**Table 1:** Previous reports of prepancreatic postduodenal portal vein

Case	Year	Author	Age	Sex	Disease	Operation	Form	Position	Anomalous branching of PV	Fragility	Complication
1	1972	Brook [8]	84	F	GB stone	Cholecystectomy	N.M	N.M	N.M.	N.M	N.M.
2	1983	Matsmoto [6]	64	M	Bile duct ca	PD	Reverse L-shaped form	Ventral to CBD	N.M.	+	–
3	1995	Matsui [9]	66	F	Bile duct ca	PD	L-shaped form	Ventral to CBD	Early bifurcation of left and right PV	+	N.M.
4	1998	Yasui [10]	65	M	Cecal ca	Rt. Hemicolectomy	L-shaped form	Ventral to CBD	Early bifurcation of left and right PV	Unclear	N.M.
5	1999	Ozeki [11]	62	F	Liver meta	Rt. hepatectomy	L-shaped form	Ventral to CBD	Early origin of P4 and right PV, P2 + 3 originated from P8	+	–
6	2000	Tanaka [12]	61	M	Bile duct ca	PD	Normal form	Parallel to CBD	N.M.	−	–
7	2003	Inoue [13]	50	M	Gastric ca	Total gastrectomy	L-shaped form	Ventral to CBD	N.M.	N.M.	–
8	2005	Jung [14]	28	F	Adenomyomatosis of GB	LC	Omega or arc-shaped form	N.M	N.M.	N.M.	–
9	2010	Tomizawa [7]	74	M	Liver meta	N.M	L-shaped form	Ventral to CBD	N.M.	N.M.	N.M.
10	2010	Tomizawa [7]	74	F	Breast ca	N.M	Normal form	Ventral to CBD	N.M.	N.M.	N.M.
11	2013	Jain [15]	56	F	Hepatitis	None	L-shaped form	Ventral to CBD	N.M.	N.M.	N.M.
12	2014	Shimizu [16]	85	F	Ampullary ca	PD	L-shaped form	Ventral to CBD	Early origin of P5	+	Massive bleeding during surgery, PVT (C-DII), ascites (C-DII)
13	2017	Goussous [17]	55	F	Choledocholithiasis	None	N.M	Parallel to CBD	N.M.	N.M.	N.M.
14	2020	Higashihara [18]	73	M	Ampullary ca	PD	L-shaped form	Ventral to CBD	Cavernous transformation	+	Massive bleeding during surgery, PVT (C-DII), DGE (C-DII)
15	2023	Present case	63	M	Hepatocellular carcinoma	Liver partial resection	L-shaped form	Ventral to CBD	Porto-portal communications	Unclear	–

*PV* portal vein, *PD* pancreatoduodenectomy, *CBD* common bile duct, *LC* laparoscopic cholecystectomy, *PVT* portal vein thrombosis, *C–D* Clavien–Dindo classification, *DGE* delayed gastric emptying, *N.M.* not mentioned

In our case, PPPV and the complex morphology of the portal vein could be identified by evaluating the contrast-enhanced CT and 3D-constructed images as preoperative examinations for HCC. His portal vein had an inverted L-shaped formation and ran along the ventral side of the bile duct, which is consistent with previous reports. In addition, the portal vein was dilated at the hepatic hilum, complicatedly meandering, bending, and branching, similar to cavernous transformation, with some porto-portal communications observed. Among the previously reported PPPV cases, the complex morphology of this case is extremely rare. We chose S8 partial resection as the operative procedure instead of anatomical resection to avoid manipulation around the hepatic hilum because of the anomaly of the portal vein and the location of the HCC on the liver surface. However, anatomical liver resection using the staining method without touching the hepatic hilum was an option to consider. At the time of resection, we considered the possibility that his portal vein wall was thinner and more fragile than usual, as pointed out by previous reports, and did not perform the Pringle maneuver, even though there was no scientific evidence that this technique was dangerous for PPPV. Fortunately, no complex meandering or irregular dilation was observed in the peripheral portal vein branches. Therefore, a partial hepatectomy for the tumor on the surface of the liver could be performed without affecting the malformation of the portal vein. If the tumor was located near the hepatic hilum, the risk of resection was thought to be markedly increased.

## Conclusion

Based on this review and our case, PPPV cases may have positional abnormalities, fragility and thinness of the portal vein wall, strong adhesion with surrounding tissue, and complicated portal branching abnormalities. Therefore, it is important to carefully identify the running and branching morphology of the portal vein on preoperative images to avoid misidentification and inadvertent damage during surgery, especially when performing surgery around the pancreatic head region, or those requiring dissection of the hepatoduodenal ligament, or major hepatectomy for patients with PPPV.

## Data Availability

All data supporting this article are included in this manuscript.

## Abbreviations

PPPV: Prepancreatic postduodenal portal vein
PDPV: Preduodenal portal vein
CT: Computed tomography
HCC: Hepatocellular carcinoma

## References

[1] Zula DJ, Houlton AY, Nataraja RM, Pacilli M (2021). Preduodenal portal vein associated with intestinal malrotation and jejunal atresia. Cureus..

[2] Thirumoorthi AS, Cowles RA (2016). Preduodenal portal vein. Surgery.

[3] Marks C (1969). Developmental basis of the portal venous system. Am J Surg.

[4] Collardeau-Frachon S, Scoazec JY (2008). Vascular development and differentiation during human liver organogenesis. Anat Rec (Hoboken).

[5] Henry BM, Skinningsrud B, Saganiak K, Pękala PA, Walocha JA, Tomaszewski KA (2019). Development of the human pancreas and its vasculature—an integrated review covering anatomical, embryological, histological, and molecular aspects. Ann Anat.

[6] Matsumoto Y, Sugahara K, Ida T, Mashimo R, Hsu KW, Fujii H (1983). Anomalies of the portal venous system: pathogenesis and its surgical implications. Jpn J Gastroenterol Surg.

[7] Tomizawa N, Akai H, Akahane M, Ino K, Kiryu S, Ohtomo K (2010). Prepancreatic postduodenal portal vein: a new hypothesis for the development of the portal venous system. Jpn J Radiol.

[8] Brook W, Gardner M (1972). Anteroposition of the portal vein and spontaneous passage of gall-stones. Case report and embryological hypothesis. Br J Surg.

[9] Matsui N, Morita T, Harada M, Morikage N, Kanazawa M, Nakamura T (1995). A case of carcinoma of the bile duct with anomaly of the portal venous system- prepancreatic postduodenal portal vein. Jpn J Gastroenterol Surg.

[10] Yasui M, Tsunoo H, Nakahara H, Asano M, Fujita H (1998). Portal vein positioned anterior to the pancreas and posterior to the duodenum-report of a case. J Jpn Surg Assoc.

[11] Ozeki Y, Tateyama K, Sumi Y, Yamada T, Yamauchi K, Bando M (1999). Major hepatectomy for liver tumor with anomalous portal branching. Jpn J Gastroenterol Surg.

[12] Tanaka K, Sano K, Yano F, Ohhira Y, Takahashi T, Suda K (2000). A case of carcinoma of the inferior bile duct with anomaly of the portal venous system-prepancreatic, postduodenal portal vein. Operation.

[13] Inoue M, Taenaka N, Nishimura S, Kawamura T, Aki T, Yamaki K (2003). Prepancreatic postduodenal portal vein: report of a case. Surg Today.

[14] Jung YJ, Lee SJ, Yang SB, Park WK, Chang JC, Kim JW (2005). Prepancreatic postduodenal portal vein: a case report. J Korean Radiol Soc.

[15] Jain VK, Rajesh S, Bhatnagar S, Dev A, Mukund A, Arora A (2013). Prepancreatic postduodenal portal vein: a rare vascular variant detected on imaging. Surg Radiol Anat.

[16] Shimizu D, Fujii T, Suenaga M, Niwa Y, Okumura N, Kanda M (2014). A case of carcinoma of the ampulla of Vater with anomaly of the portal venous system: prepancreatic postduodenal portal vein. Jpn J Gastroenterol Surg.

[17] Goussous N, Cunningham SC (2017). Prepancreatic postduodenal portal vein: a case report and review of the literature. J Med Case Rep.

[18] Higashihara T, Morita Y, Hayashi T, Takahashi M, Yogi N, Sasaki S (2022). Hepatobiliary-pancreatic surgery for patients with a prepancreatic postduodenal portal vein: a case report and literature review. BMC Surg.

